# Supplementary material for: MetaRibo-Seq measures translation in microbiomes
Source: Nat Commun. 2020 Jun 29;11:3268. doi: 10.1038/s41467-020-17081-z (PMC7324362; doi:10.1038/s41467-020-17081-z)
Supplement: Supplementary file 10 — Supplementary Data 7 [file 41467_2020_17081_MOESM10_ESM.zip › File2/Confidence_High_Taxonomy/88664_out.krona.html]

Javascript must be enabled to view this page.

members
magnitude
magnitudeUnassigned
count
unassigned
taxon
rank

88664\_out

2

superkingdom
2
2

phylum
1239
2

186801
class
1

186802
order
1

186803
family
1

43994
genus
1

43995
species

SRS104842\_contig\_number\_contig-100\_2051.346230
1

91061
class
1

order
186826
1

186828
family
1

genus
117563
1

species
1581071

SRS017139\_contig\_number\_25131
1
